# Nodal Burden and Oncologic Outcomes in Patients With Residual Isolated Tumor Cells After Neoadjuvant Chemotherapy (ypN0i+): The OPBC-05/ICARO Study

**DOI:** 10.1200/JCO.24.01052

**Published:** 2024-11-07

**Authors:** Giacomo Montagna, Alison Laws, Massimo Ferrucci, Mary M. Mrdutt, Susie X. Sun, Suleyman Bademler, Hakan Balbaloglu, Nora Balint-Lahat, Maggie Banys-Paluchowski, Andrea V. Barrio, John Benson, Nuran Bese, Judy C. Boughey, Marissa K. Boyle, Emilia J. Diego, Claire Eden, Ruth Eller, Maite Goldschmidt, Callie Hlavin, Martin Heidinger, Justyna Jelinska, Güldeniz Karadeniz Cakmak, Susan B. Kesmodel, Tari A. King, Henry M. Kuerer, Julie Loesch, Francesco Milardi, Dawid Murawa, Tracy-Ann Moo, Tehillah S. Menes, Daniele Passeri, Jessica M. Pastoriza, Andraz Perhavec, Nina Pislar, Natália Polidorio, Avina Rami, Jai Min Ryu, Alexandra Schulz, Varadan Sevilimedu, M. Umit Ugurlu, Cihan Uras, Annemiek van Hemert, Stephanie M. Wong, Tae-Kyung Robyn Yoo, Jennifer Q. Zhang, Hasan Karanlik, Neslihan Cabioğlu, Marie-Jeanne Vrancken Peeters, Monica Morrow, Walter P. Weber, on behalf of the ICARO Study Group, ICARO Study Group

**Affiliations:** ^1^ Department of Surgery, Memorial Sloan Kettering Cancer Center, New York, NY; ^2^ Division of Breast Surgery, Department of Surgery, Brigham and Women's Hospital, Boston, MA; ^3^ Veneto Institute of Oncology, Padua, Italy; ^4^ Division of Breast and Melanoma Surgical Oncology, Department of Surgery, Mayo Clinic, Rochester, MN; ^5^ Department of Breast Surgical Oncology, The University of Texas MD Anderson Cancer Center, Houston, TX; ^6^ Institute of Oncology, Istanbul University, Istanbul, Turkey; ^7^ Department of General Surgery, School of Medicine, Zonguldak Bulent Ecevit University, Zonguldak, Turkey; ^8^ Sheba Medical Center, Tel Hashomer, Israel; ^9^ Faculty of Medicine, Tel Aviv University, Tel Aviv, Israel; ^10^ University Hospital Schleswig-Holstein Campus Lübeck, Lübeck, Germany; ^11^ Cambridge University Hospital NHS Foundation Trust, Cambridge, United Kingdom; ^12^ Research Institute of Senology Acibadem, Istanbul, Turkey; ^13^ Cedars-Sinai Medical Center, Los Angeles, CA; ^14^ University of Pittsburgh Medical Center, Pittsburgh, PA; ^15^ Department of Surgery, New York-Presbyterian/Weill Cornell Medical Center, New York, NY; ^16^ University of Basel, Basel, Switzerland; ^17^ Breast Center, University Hospital Basel, Basel, Switzerland; ^18^ General Surgery and Surgical Oncology Clinic, Collegium Medicum, University Zielona Gora, Zielona Góra, Poland; ^19^ DeWitt Daughtry Department of Surgery, University of Miami/Sylvester Comprehensive Cancer Center, Miami, FL; ^20^ Breast Surgery Division, Department of Surgery, Montefiore Medical Center, Montefiore Einstein Center for Cancer Care, New York, NY; ^21^ Department of Surgical Oncology, Institute of Oncology Ljubljana, Ljubljana, Slovenia; ^22^ Department of Surgery, Samsung Medical Center, Sungkyunkwan University School of Medicine, Seoul, Republic of Korea; ^23^ Department of Clinical Research, University Hospital Basel, Basel, Switzerland; ^24^ Department of Epidemiology and Biostatistics, Memorial Sloan Kettering Cancer Center, New York, NY; ^25^ Marmara University School of Medicine, Istanbul, Turkey; ^26^ Department of Surgery, Stichting HET Netherlands Kanker Instituut-Antoni van Leeuwenhoek, Amsterdam, the Netherlands; ^27^ McGill University Medical School, Montreal, QC, Canada; ^28^ Department of Surgery, Asan Medical Center, University of Ulsan College of Medicine, Seoul, Republic of Korea; ^29^ Department of Surgery, University of Pennsylvania Health System, Philadelphia, PA; ^30^ Breast Unit, Department of General Surgery, Istanbul University Istanbul Faculty of Medicine, Istanbul, Turkey; ^31^ Department of Surgery, Amsterdam University Medical Center, Amsterdam, the Netherlands

## Abstract

**PURPOSE:**

The nodal burden of patients with residual isolated tumor cells (ITCs) in the sentinel lymph nodes (SLNs) after neoadjuvant chemotherapy (NAC) (ypN0i+) is unknown, and axillary management is not standardized. We investigated rates of additional positive lymph nodes (LNs) at axillary lymph node dissection (ALND) and oncologic outcomes in patients with ypN0i+ treated with and without ALND.

**METHODS:**

The Oncoplastic Breast Consortium-05/ICARO cohort study (ClinicalTrials.gov identifier: NCT06464341) retrospectively analyzed data from patients with stage I to III breast cancer with ITCs in SLNs after NAC from 62 centers in 18 countries. The primary end point was the 3-year rate of any axillary recurrence. The rate of any invasive recurrence was the secondary end point.

**RESULTS:**

In total, 583 patients were included, of whom 182 (31%) had completion ALND and 401 (69%) did not. The median age was 48 years. Most patients (74%) were clinically node-positive at diagnosis and 41% had hormone receptor–positive/human epidermal growth factor receptor 2–negative tumors. The mean number of SLNs with ITCs was 1.2. Patients treated with ALND were more likely to present with cN2/3 disease (17% *v* 7%, *P* < .001), have ITCs detected on frozen section (62% *v* 8%, *P* < .001), have lymphovascular invasion (38% *v* 24%, *P* < .001), and receive adjuvant chest wall (89% *v* 78%, *P* = .024) and nodal radiation (82% *v* 75%, *P* = .038). Additional positive nodes were found at ALND in 30% of patients, but only 5% had macrometastases. The 3-year rates of any axillary and any invasive recurrence were 2% (95% CI, 0.95 to 3.6) and 11% (95% CI, 8 to 14), respectively, with no statistical difference by type of axillary surgery.

**CONCLUSION:**

The nodal burden in patients with ypN0(i+) was low, and axillary recurrence after ALND omission was rare in patients selected for this approach. These results do not support routine ALND in all patients with ypN0(i+).

## INTRODUCTION

In the upfront surgery setting, volume of disease in the sentinel lymph nodes (SLNs) is an important predictor of the likelihood of additional non-SLN metastases at axillary lymph node dissection (ALND).^1-5^ In the setting of neoadjuvant chemotherapy (NAC), patients with positive SLNs have higher residual nodal burden than patients with a positive SLN treated with upfront surgery, irrespective of the size of the nodal metastasis and tumor subtype.^6-9^ Therefore, ALND is the current standard of care for residual micrometastatic and macrometastatic disease after NAC.^10-12^

**CONTEXT**

**Key Objective**
To investigate the role of axillary lymph node dissection (ALND) in patients with residual isolated tumor cells (ITCs) in the sentinel lymph nodes (SLNs) after neoadjuvant chemotherapy.
**Knowledge Generated**
In this cohort study of 583 patients with residual ITCs in the SLNs, additional positive lymph nodes were found at ALND in 30% of patients but contained macrometastases in only 5%. Axillary recurrence was rare in patients selected for omission of ALND.
**Relevance *(K.D. Miller)***
These results are similar to those in patients undergoing primary surgery and clearly indicate that ITCs should not affect the management of the axilla. ALND should be limited to patients with locally advanced disease with gross nodal involvement.*
*Relevance section written by *JCO* Senior Deputy Editor Kathy D. Miller, MD.

Residual isolated tumor cells (ITCs) after NAC are found in approximately 1.5% of all patients undergoing NAC.^13^ The likelihood of finding additional positive lymph nodes (LNs) at ALND and the optimal management of the axilla in these patients are currently unclear.^6,13-19^ Despite lack of consensus on the oncologic safety of omitting ALND among this group, patterns of care studies suggest increasing adoption of this approach.^10,19^ Given the lack of forthcoming prospective studies, this study assimilated real-world data from a large international cohort with the aim of determining the likelihood of non-SLN involvement in patients with ITCs in the SLNs and assessed clinical outcomes by use of ALND.

## METHODS

### Study Population

The Oncoplastic Breast Consortium (OPBC)-05/ICARO cohort study (ClinicalTrials.gov identifier: NCT06464341) retrospectively analyzed institutional databases from 62 cancer centers located in 18 countries (the majority of centers are within the OPBC network). Institutional review board approval was obtained for each site in the United States, with informed consent waived due to use of deidentified data. At sites outside of the United States, informed consent was sought according to ethical approval that was study-/database-specific or based on general consent, according to site-specific and national standards. A data use agreement was executed between Memorial Sloan Kettering Cancer Center (MSKCC) and the other North American institutions, and between MSKCC and the University Hospital of Basel, Switzerland, which served as the coordinating center to collect data from all OPBC sites. The study followed the Strengthening the Report of Observational Studies in Epidemiology guidelines for reporting observational studies.^20^ The principal investigator at each site was responsible for data collection, curacy, and transfer, whereas data cleaning and analysis were conducted at MSKCC.

Patients with clinical T1-4 N0-3 breast cancer at diagnosis treated with NAC between March 2008 and May 2022 who were found to have ITCs only (clusters of tumor cells ≤0.2 mm or a cluster of <200 cells in a single cross-sectional image) at frozen section (FS) or on final paraffin sections determined by sentinel lymph node biopsy (SLNB), targeted axillary dissection (TAD), or the marking axillary lymph nodes with radioactive iodine seeds (MARI) procedure were selected. The study was inclusive of both high-volume centers and small breast units in the private, public, and academic settings. Downstaging to clinically N0 was required for patients who presented with palpable nodal disease. Patients with inflammatory breast cancer, stage IV disease at presentation, who had ALND as a primary procedure, and who received neoadjuvant endocrine therapy were excluded. Those with micrometastases or macrometastases in any SLNs at FS or final pathology were ineligible. We excluded One Step Nucleic Acid Amplification technology due to the absence of standardization.

### Surgical Technique

The SLNB procedure included removal of all LNs that were either blue (isosulfan blue dye, methylene blue) or radioactive (technetium Tc 99m). Palpably abnormal nodes were considered SLNs. For cN0 patients at presentation, single tracer was allowed, whereas for cN+ patients, use of dual-tracer mapping was mandatory. TAD consisted of SLNB with single- or dual-tracer mapping plus image-guided localization of the initially biopsy-proven and clipped node. The MARI procedure consisted of selective removal of the pathologically proven metastatic LN, which was marked with an iodine marker before NAC. Details of the surgical procedures specific to each site are provided in the Data Supplement (online only).

### Pathology Assessment

Details regarding pathology assessment of the SLNs (FS, quantification of the volume of disease, and use of immunohistochemical staining) specific to each institutions are provided in the Data Supplement.

### Systemic and Radiation Therapy

NAC regimens, adjuvant systemic therapy, and regional nodal irradiation (RNI) were administered in accordance with national guidelines. Radiation dose, schedule, and treatment fields specific to each center are provided in the Data Supplement.

### End Points

The primary end point was the 3-year rate of axillary recurrence (isolated or combined with local and distant recurrence within 30 days). Secondary end points included the 3-year rate of isolated axillary recurrence and the 3-year rate of any invasive recurrence, be this locoregional or distant. The proportion of additional positive LNs at ALND was also assessed.

### Statistical Analysis

Clinicopathologic and demographic characteristics were compared between surgical groups using the Wilcoxon rank-sum test or *t* test for continuous variables, and the chi-square or Fisher's exact test for categorical variables. The rate of additional positive LNs at ALND was compared between cN0 and cN+ patients at presentation using a chi-square test. Cumulative incidence of axillary recurrence and any invasive recurrence (locoregional or distant) was assessed using a competing risk analysis (Data Supplement). Follow-up data were obtained from the date of surgery. Three-year cumulative incidence rates were compared between patients treated with and without ALND using Gray's test. A *P* < .05 was considered statistically significant. Statistical analysis was performed using R 4.3.2 (R Core Development Team, Vienna, Austria).

## RESULTS

### Patient and Treatment Characteristics

**FIG 1.**
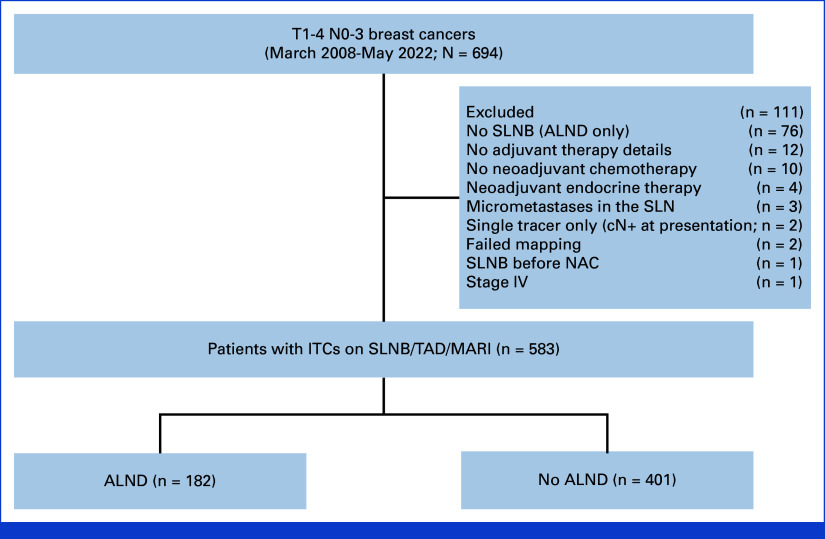
Flow diagram. ALND, axillary lymph node dissection; ITC, isolated tumor cell; MARI, marking axillary lymph nodes with radioactive iodine seeds; NAC, neoadjuvant chemotherapy; SLN, sentinel lymph node; SLNB, sentinel lymph node biopsy; TAD, targeted axillary dissection.

Data were collected from a total of 694 patients, with 111 excluded due to failure to meet inclusion criteria (Fig 1). The study population included 583 patients with residual ITCs detected on SLNB, TAD, or MARI, among whom 31.2% (n = 182) were treated with ALND and 68.8% (n = 401) without. The baseline characteristics of the cohort, stratified by surgical group, are listed in Table 1. The median age was 48 (IQR, 41-57) years, and most patients had clinical (c) T2 tumors (57%) and were clinically node-positive (cN+) at presentation (74%). Fewer than half of tumors (41%) were hormone receptor–positive (HR+)/human epidermal growth factor receptor 2–negative (HER2–), 38% were HER2-positive, and the remainder (21%) triple-negative. More than three quarters (77%) of patients received adjuvant RNI. Patients treated with ALND were more likely to present with cN2/3 disease (17% *v* 7%, *P* < .001), have ITCs detected on FS (62% *v* 8%, *P* < .001), have lymphovascular invasion (38% *v* 24%, *P* < .001), and receive chest wall irradiation (89% *v* 78%, *P* = .024) and RNI (82% *v* 74%, *P* = .038). Conversely, this group was less likely to have a lobular phenotype (4.3% *v* 11%, *P* = .021).

**TABLE 1.** Clinicopathologic Features of the Study Cohort, Stratified by Surgical Group

Feature	Overall (n = 583)	No ALND (n = 401)	ALND (n = 182)	*P* ^a^
Age, years (IQR)	48 (41-57)	49 (40-57)	49 (43-58)	.11
Race/ethnicity, No. (%)				.5
Asian	64 (11)	40 (10)	24 (13)	
Black	27 (4.6)	22 (5.5)	5 (2.7)	
Hispanic	31 (5.3)	23 (5.7)	8 (4.4)	
White	447 (77)	306 (76)	141 (77)	
Other/unknown	14 (2.4)	10 (2.5)	4 (2.2)	
Clinical T stage at presentation, No. (%)				.15
1	95 (16)	68 (17)	27 (15)	
2	332 (57)	219 (55)	113 (62)	
3	136 (23)	102 (25)	34 (19)	
4	19 (3.3)	12 (3.0)	7 (3.8)	
X	1 (0.2)	0 (0)	1 (0.5)	
Clinical N stage at presentation, No. (%)				**<.001**
0	150 (26)	120 (30)	30 (16)	
1	376 (64)	254 (63)	122 (67)	
2	44 (7.5)	21 (5.2)	23 (13)	
3	13 (2.2)	6 (1.5)	7 (3.8)	
Tumor subtype, No. (%)				.6
HR+/HER2–	240 (41)	161 (40)	79 (43)	
HR+/HER2+	161 (28)	109 (27)	52 (29)	
HR–/HER2+	60 (10)	41 (10)	19 (10)	
HR–/HER2–	122 (21)	90 (22)	32 (18)	
Histology, No. (%)				**.021**
Ductal	516 (89)	350 (87)	166 (91)	
Lobular or mixed	53 (9.1)	44 (11)	9 (4.3)	
Other	14 (2.4)	7 (1.7)	7 (3.8)	
Tumor differentiation, No. (%)				.13
Well	34 (6.4)	29 (7.8)	5 (3.1)	
Moderately	210 (39)	146 (39)	64 (40)	
Poorly	290 (54)	199 (53)	91 (57)	
Unknown	49	27	22	
LVI, No. (%)				**<.001**
Present	167 (29)	97 (24)	70 (38)	
Type of breast surgery, No. (%)				.13
BCS	267 (46)	192 (48)	75 (41)	
Mastectomy	316 (54)	209 (52)	107 (59)	
Breast pCR (ypT0/is), No. (%)				.8
Yes	162 (28)	110 (27)	52 (29)	
NAC regimen HER2– (n = 362), No. (%)				.8
AC-T	287 (79)	197 (78)	90 (81)	
AC-T + Carbo	24 (6.6)	15 (6)	9 (8.1)	
AC-T + Carbo + pembrolizumab	10 (2.8)	7 (2.8)	3 (2.7)	
Anthracycline-free regimen^b^	10 (2.8)	8 (3.2)	2 (1.8)	
Other	31 (8.6)	24 (9.6)	7 (6.3)	
NAC regimen HER2+ (n = 221), No. (%)				.068
AC-TH	50 (22.5)	31 (20.7)	19 (27)	
AC-THP	65 (29)	43 (29)	22 (31)	
TCH	4 (1.8)	2 (1.3)	2 (2.8)	
TCHP	67 (30)	54 (36)	13 (18)	
Other	35 (16)	20 (13)	15 (21)	
Axillary staging technique in cN+ (n = 433), No. (%)				**<.001**
SLNB with dual-tracer mapping	251 (58)	146 (52)	105 (69)	
TAD	147 (34)	104 (37)	43 (28)	
MARI	35 (8.1)	31 (11)	4 (2.6)	
SLNs removed, No. (mean, SD)^c^	3.3 (0, 16)	3.5 (1, 16)	2.8 (0, 10)	**<.001**
Non-SLNs removed, No. (mean, SD)	0.8 (1.52)	0.8 (1.46)	0.7 (1.64)	**.015**
SLNs with ITCs, No. (mean, SD)	1.2 (0, 6)	1.2 (0, 6)	1.2 (0, 6)	.6
Total lymph nodes removed, No. (mean, SD)	7 (1, 37)	4 (1, 16)	15 (4, 37)	**<.001**
ITCs detected on frozen section, No. (%)				**<.001**
Yes	139 (25)	31 (7.9)	108 (62)	
Not performed/unknown	20	11	9	
Breast RT (n = 267), No. (%)	262 (98)	187 (97)	75 (100)	.3
Chest wall RT (n = 316), No. (%)	259 (82)	164 (78)	95 (89)	**.024**
Nodal RT, No. (%)	447 (77)	298 (74)	149 (82)	**.038**
Adjuvant chemotherapy,^d^ No. (%)	92 (16)	60 (15)	32 (18)	.4
Adjuvant endocrine therapy (n = 401), No. (%)	379 (95)	258 (96)	121 (92)	.2
Adjuvant anti-HER2 therapy (n = 221), No. (%)	211 (95)	142 (95)	69 (97)	.5

NOTE. Data are expressed as frequency (column percentage) for categorical variables, and median (IQR) or mean (SD) for continuous variables. Statistically significant values are indicated in bold.

Abbreviations: AC-T, doxorubicin hydrochloride (doxorubicin) and cyclophosphamide, followed by paclitaxel (taxol); AC-TH, doxorubicin hydrochloride and cyclophosphamide followed by paclitaxel or docetaxel and trastuzumab; AC-THP, doxorubicin hydrochloride and cyclophosphamide followed by paclitaxel or docetaxel, trastuzumab, and pertuzumab; ALND, axillary lymph node dissection; BCS, breast-conserving surgery; Carbo, carboplatin; H, herceptin; HER2, human epidermal growth factor receptor 2; HR, hormone receptor; ITC, isolated tumor cell; LVI, lymphovascular invasion; MARI, marking axillary lymph nodes with radioactive iodine seeds; NAC, neoadjuvant chemotherapy; pCR, pathologic complete response; RT, radiation therapy; SD, standard deviation; SLN, sentinel lymph node; SLNB, sentinel lymph node biopsy; TAD, targeted axillary dissection; TCH, docetaxel, carboplatin, and trastuzumab; TCHP, docetaxel or paclitaxel, carboplatin, trastuzumab, and pertuzumab.

^a^ Results are from the Wilcoxon rank-sum test for continuous variables, and Fisher's exact test or the chi-square test of independence for categorical variables.

^b^ Includes cyclophosphamide, methotrexate, and fluorouracil; and taxotere and cyclophosphamide.

^c^ MARI participants were excluded (n = 35).

^d^ Capecitabine was the most common type of adjuvant chemotherapy (75%).

### Axillary Staging Characteristics

Among clinically node-positive patients, axillary staging was performed with SLNB, TAD, and the MARI technique in 58%, 34%, and 8% of patients, respectively. Patients who underwent TAD and MARI were less likely to undergo ALND compared with patients who underwent SLNB (26% *v* 42%, *P* < .001). Significantly fewer SLNs were removed in the ALND group compared with the non-ALND group (mean number of nodes 2.8 *v* 3.5, respectively; *P* < .001). However, the mean number of SLNs with ITCs was the same in both surgical groups (n = 1.2; Table 1). The practice of ALND did not change over the study period when comparing patients treated before and after 2019 (*P* > .9).

### Nodal Burden in Patients Undergoing ALND

**FIG 2.**
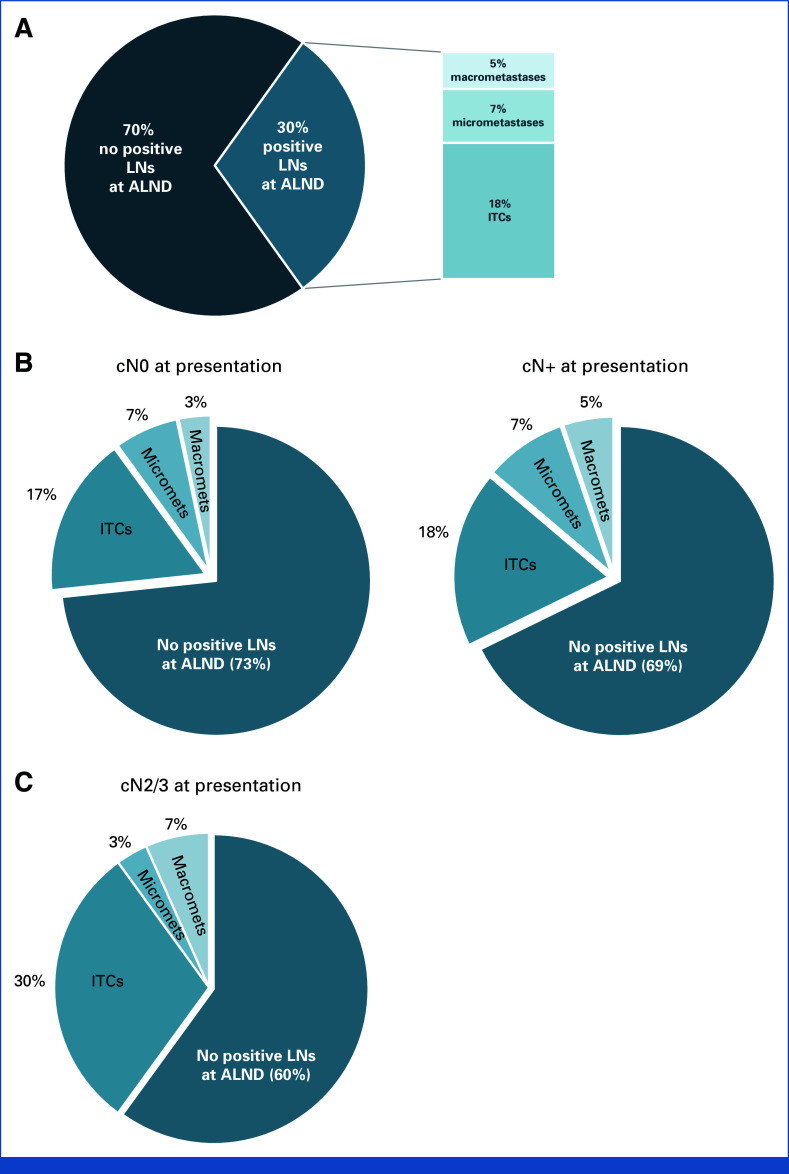
Proportion of patients with additional positive LNs at ALND by (A) all patients undergoing ALND (n = 182); (B) stratified by clinical nodal status at presentation (cN0 [n = 30] *v* cN+ [n = 152]); and (C) cN2/3 patients (n = 57). ALND, axillary lymph node dissection; LN, lymph node.

In the ALND group (n = 182), additional positive nodes were found in 55 (30%) patients, consisting of ITCs in 32 (18%), micrometastases in 13 (7%), and macrometastases in nine patients (5%; Fig 2A). Among the 32 patients with additional ITCs, the number of additional positive LNs was one in 18 (56%), two in seven (22%), three in five (16%), and ≥4 in two (6%) patients. Among the 13 patients with additional micrometastases, the number of additional positive LNs was one in three (23%), two in two (15%), three in four (31%), and ≥4 in four (31%) patients. Finally, among the nine patients with additional macrometastases, the number of additional positive LNs was one in five (56%), two in three (33%), and ≥4 in one (11%) patient. When stratified by clinical nodal stage at presentation, there was no statistically significant difference in the rate of positive nodes at ALND between patients who were cN0 and cN+ (27% *v* 31%, respectively, *P* = .6). The distribution of ITCs, micrometastases, and macrometastases in nonsentinel nodes was also similar among the two groups (Fig 2B). Among 57 patients with cN2/3 disease at presentation, 30 (53%) underwent an ALND. Additional positive LNs were found in 12 (40%) of 30 patients and consisted of additional ITCs in nine (30%), micrometastases in one (3%), and macrometastases in two patients (7%; Fig 2C).

### Oncologic Outcomes in Patients Treated With and Without ALND

**FIG 3.**
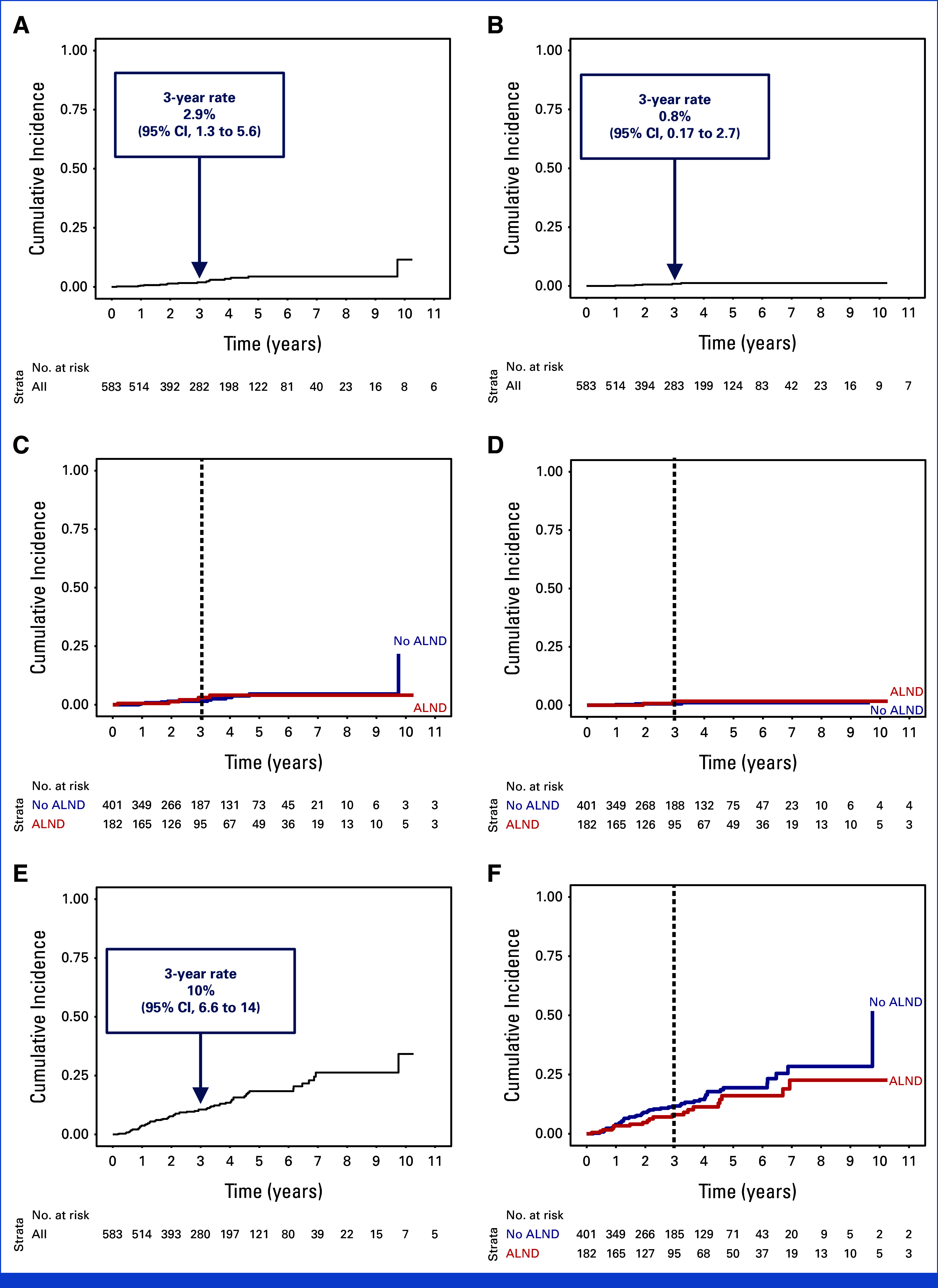
Competing risk analysis for (A) any axillary recurrence (overall cohort); (B) isolated axillary recurrence (overall cohort); (C) any axillary recurrence (stratified by surgical group); (D) isolated axillary recurrence (stratified by surgical group); (E) any invasive recurrence (overall cohort); and (F) any invasive recurrence (stratified by surgical group). ALND, axillary lymph node dissection.

The median follow-up for the entire cohort was 3.2 years (IQR, 1.8-4.9) and was comparable between the ALND (3.4 years, IQR, 1.9-5.6) and non-ALND (3.1 years, IQR, 1.8-4.8) groups. During the study period, there were five isolated axillary recurrences (two in the ALND group and three in the non-ALND group) and nine synchronous locoregional and distant recurrences (three in the ALND group, six in the non-ALND group). The 3-year rate of any axillary recurrence (isolated or combined with local or distant recurrence) for the entire cohort was 2.0% (95% CI, 0.95 to 3.6; Fig 3A), whereas the 3-year rate of isolated axillary recurrence was 0.58% (95% CI, 0.12 to 2.0; Fig 3B). Moreover, there were no statistically significant differences between patients treated with and without ALND for 3-year rates of any and isolated axillary recurrence (1.5% *v* 3.1%; *P* = .8 and 0.58% *v* 1.7%; *P* = .7), respectively (Figs 3C and 3D). The 3-year rate of any invasive (locoregional or distant) recurrence in the entire cohort was 11% (95% CI, 8 to 14; Fig 3E), with no significant difference in outcome between patients treated with and without ALND (8.1% *v* 12%, *P* = .13; Fig 3F). Exploratory 5-year results were as follows: the 5-year rate of any and isolated axillary recurrence was 4.4% (95% CI, 2.5 to 7.2) and 1.3% (95% CI, 0.47 to 2.9), respectively (with no significant differences between groups [ALND *v* no-ALND], 4.1% *v* 4.6%; *P* = .8 and 1.7% *v* 1.1%; *P* = .7, respectively), and the 5-year rate of any invasive recurrence was 18% (95% CI, 14 to 23; with no significant difference between groups [ALND *v* no-ALND], 16% *v* 19%; *P* = .13).

## DISCUSSION

Patients with a positive SLN after NAC have a higher axillary nodal burden than patients with a positive SLN in the upfront surgery setting.^6,7,9^ Moreover, NAC patients were not included in earlier trials evaluating omission of ALND (with or without RNI) for patients with limited nodal disease on SLNB.^21-23^ Axillary management in the setting of residual ITCs is largely non–evidence-based, and reliant upon clinical opinion and physician discretion. Uncertainty relates not just to the unknown nonsentinel nodal burden but also to the biologic significance of ITCs after axillary downstaging in response to NAC.

In this cohort of 583 patients with ITCs detected on SLNB, TAD, or MARI, additional positive non-SLNs were found in 30% of the 182 ALND patients, with no statistically significant difference by clinical nodal status at presentation. Importantly, more than half of the patients with additional nodal disease involved ITCs only, with macrometastases and micrometastases found in only 5% and 7% of patients, respectively. The present results confirm previous findings from both the SN-FNAC and ACOSOG Z1071 trials evaluating feasibility of SLNB after NAC for biopsy-proven nodal disease, which reported that four (57%) of seven and four (36%) of 11 patients with ITCs had additional positive nodes at ALND, respectively. Similarly, Moo et al^6^ found that one (17%) of six patients with ITCs had additional positive LNs at ALND.

We observed that completion ALND was omitted in more than two thirds of patients with no significant change in surgical practice over the study period. This accords with previous studies reporting ALND omission in a significant proportion of patients with low-volume residual disease after NAC. In a study from the American Cancer Society/American College of Surgeons National Cancer Database, Wong et al^24^ found that among 12,965 women treated with NAC between 2012 and 2015, 37% of patients with residual ITCs and 24% of patients with micrometastases did not undergo completion ALND. Our study is the first to support the safety of ALND omission in patients with residual ITCs; indeed, there appears to be no oncologic detriment, with no significant differences in key end points of clinical outcome, between patients treated with and without ALND.

Clinicopathologic factors, known before or during surgery, associated with performing an ALND included higher nodal stage at presentation (assessed by clinical examination and imaging) and detection of ITCs on FS.

As the evidence to support the feasibility and accuracy of SLNB in patients with cN2/3 is limited (the validation studies of SLNB after NAC only included a small proportion of patients with cN2 disease and did not include cN3 patients)^14,15,25,26^ and as prospective data on the safety of ALND omission after downstaging with NAC in this population are lacking, this is to be expected. Until more data from prospective studies become available,^27^ ALND remains the standard of care for patients presenting with locally advanced breast cancer.

As anticipated, only a minority of patients (25%) in this study were detected on FS, since the size of a nodal metastasis is directly proportional to its probability of being identified on FS, making detection of ITCs challenging.^28,29^ Interestingly, those patients who underwent ALND were more likely to have ITCs detected on FS than those who did not (62% *v* 8%, respectively), suggesting that most surgeons are unlikely to perform second surgery for low-volume disease.

In patients with residual micrometastases and macrometastases on SLNB, omission of ALND was not associated with any differences in the use of postneoadjuvant systemic therapy.^30^ In the setting of residual breast disease, the presence of ITCs is unlikely to alter subsequent systemic therapy decisions. However, in patients who achieve a breast pCR and have residual nodal ITCs, the presence of additional nodal disease removed by ALND may indeed prompt a recommendation change, particularly in HER2+ and triple-negative tumors. In this study, among patients with HER2+ tumors and triple negative breast cancer who had an ALND and achieved a breast pCR (n = 33), all those with additional positive non-SLNs (n = 9) had low-volume disease (in seven patients, the non-SLNs contained ITCs, and in two patients they contained micrometastases). Another important question is whether omitting ALND would preclude eligibility for treatment with a cyclin-dependent kinase (CDK) 4/6 inhibitor on the basis of the total number of positive LNs.^31^ Of the 80 patients with HR+/HER2– undergoing ALND, five (6%) of 80 had ypN2 disease, but only one patient would have not been eligible to receive a CDK 4/6 inhibitor on the basis of other high-risk features at presentation (tumor size or grade).

Overall, these results should reassure medical oncologists that ALND omission in this population would not negatively affect systemic therapy recommendations. Similar results were recently reported in a prospective study of ALND omission in node-positive patients, treated in both adjuvant and neoadjuvant settings, which found that omitting ALND was not associated with differences in systemic therapy recommendations.^30^

It is important to note that the majority of patients (77% overall, 74% with no ALND) in this cohort received RNI. In four of the five patients who experienced an isolated axillary recurrence, nodal RT was omitted, suggesting that RNI contributed to the extremely low rate of axillary failure observed in this cohort. Because of the small number of events and the inability to account for the random variation present at the single-institution level, we were not able to assess the independent effect of RNI on nodal recurrence. However, recent results from a time-driven analysis of the NSABP-B51 trial, which randomly assigned node-positive patients who were found to be ypN0/i+ at surgery to RNI versus no RNI, found no benefit of RNI for either the primary end point of invasive recurrence-free survival or the secondary end points of locoregional-free survival or distant recurrence-free survival (overall survival data immature due to fewer recurrence events than expected).^32^ Although the exact number of patients with ITCs included was not reported (ypN0 category included ITCs) and the median follow-up is short (59.5 months), these data suggest that locoregional therapy in patients with ypN0(i+) may be further de-escalated.

Although immunohistochemistry (IHC) is not routinely used in all centers for SLN examination post-NAC, it can increase detection of small tumor foci in SLNs. In the SN-FNAC trial, for example, all patients with ITCs (n = 6) were detected by IHC,^15^ although the likelihood of detecting occult macrometastases by IHC is very low.^33^ As the presence of residual ITCs does not appear to influence surgical and adjuvant treatment decisions for the great majority of patients, results of this study argue against routine use of IHC for analysis of SLNs after NAC.

To our knowledge, this is the first study to compare outcomes in patients with residual ITCs treated with and without ALND, and to evaluate the residual nodal burden in this patient population. Our study, however, has several limitations, mainly related to its retrospective design, the real-world origin of the data, and selection bias, as the decision to omit ALND was based on lower baseline risk as well as surgeon, and perhaps patient, choice. Although we demonstrated excellent oncologic outcomes, the selection bias limited direct comparisons between patients treated with and without ALND. However, to the authors' knowledge, high-level evidence to address this clinical question is not expected. Patients with residual ITCs only were excluded from the ALLIANCE A011202 trial, which evaluates omission of ALND in favor of RNI in patients with ypN+. Although patients with residual ITCs are eligible for the ongoing TAXIS trial, whereby ALND is omitted as part of tailored axillary surgery, only very few patients with ypN0(i+) have been included.^8,34^ In the absence of level 1 evidence to guide routine clinical care, the present real-world study was performed to reduce surgical overtreatment. A second limitation of our study is that despite the pooled analysis from 62 centers, the determination of the sample size was pragmatic and based on the number of patients available at the participating sites. A post hoc power analysis showed that a 10-times larger sample size would have been necessary to achieve adequate power to detect the observed difference in cumulative incidence rates. A third limitation of our study is its relatively short median follow-up of 3.2 years. Although longer follow-up is planned, based on data from the upfront surgery setting, axillary recurrence is an early event, with most events occurring within 5 years.^21,35^ It is therefore anticipated that these findings will be reaffirmed with more prolonged follow-up. Other limitations include lack of standardized pathologic assessment and centralized review, and a low number of events, which precludes an adjustment for baseline characteristics and location-based differences.

In conclusion, the likelihood of finding additional positive LNs on completion ALND for residual ITCs in the SLN after NAC was lower than for patients with residual micrometastases and macrometastases, with additional macrometastases found in only 5% of patients. Rates of axillary and invasive recurrence were low in patients selected for ALND omission on the basis of lower clinical risk at baseline and increased use of nodal RT. Overall, these results do not support routine ALND in patients with residual ITCs after NAC, thereby questioning the routine use of IHC staining for SLN examination after NAC.

## APPENDIX 1. The ICARO Study Group

Sung Gwe Ahn, MD, PhD; Mariacarla Andreozzi, PhD; Daniel Meirelles Barbalho, MD, PhD; Jean François Boileau, MD, MSc; Edi Brogi, MD, PhD; Flavia Vidal Cabero, MD, MSc; Daniela Cocco, MD; Fabio Corsi, MD; Angelena Crown, MD; Eelco de Bree, MD, PhD; Maria del Mar Vernet-Tomas, MD, PhD; Christine Deutschmann, MD; Nina Ditsch, MD; Emanuela Esposito, MD, PhD; Oluwadamilola M. Fayanju, MD, MPHS; Franziska Fick, MD; Florian Fitzal, MD; Meghan R. Flanagan, MD, MPH; Damiano Gentile, MD; Oreste D. Gentilini, MD; Anne Grabenstetter, MD; Mehmet Ali Gulcelik, MD; Jörg Heil, MD, PhD; Johannes Holtschmidt, MD; Natalia Krawczyk, MD, PhD; Thorsten Kühn, MD, PhD; Sherko Kümmel, MD, PhD; Cornelia Leo, MD; Mahmut Muslumanoglu, MD; Valentina Nekljudova, PhD; Lisa A. Newman, MD, MPH; Melissa L. Pilewskie, MD; Fiorita Poulakaki, MD, PhD; Fabian Riedel, MD; Nicola Rocco, MD, PhD; Freya R. Schnabel, MD; Christopher Schwartz, DO; Emily L. Siegel, MD; Colin Simonson, MD; Christian Singer, MD, PhD; Leonardo Ribeiro Soares, MD, PhD; Ekaterini Christina Tampaki, MD, PhD; Athanasios Tampakis, MD, PhD; Marios Konstantinos Tasoulis, MD, PhD; Christoph Tausch, MD; Cicero Urban, MD, PhD; Astrid Botty Van den Bruele, MD; Glenn Vergauwen, MD, PhD; Denise Vorburger, MD; Fredrik Wärnberg, MD; Anna Weiss, MD; Austin D. Williams, MD, MSEd; Kerstin Wimmer, MD, PhD; Steven G. Woodward, MD; Firdos M. Ziauddin, MD

## References

[1] Chu KU, Turner RR, Hansen NM, et al: Do all patients with sentinel node metastasis from breast carcinoma need complete axillary node dissection? Ann Surg 229:536-541, 1999 10203087 10.1097/00000658-199904000-00013 PMC1191740

[2] Cserni G, Gregori D, Merletti F, et al: Meta-analysis of non-sentinel node metastases associated with micrometastatic sentinel nodes in breast cancer. Br J Surg 91:1245-1252, 2004 15376203 10.1002/bjs.4725

[3] Kamath VJ, Giuliano R, Dauway EL, et al: Characteristics of the sentinel lymph node in breast cancer predict further involvement of higher-echelon nodes in the axilla: A study to evaluate the need for complete axillary lymph node dissection. Arch Surg 136:688-692, 2001 11387010 10.1001/archsurg.136.6.688

[4] Turner RR, Chu KU, Qi K, et al: Pathologic features associated with nonsentinel lymph node metastases in patients with metastatic breast carcinoma in a sentinel lymph node. Cancer 89:574-581, 2000 10931456 10.1002/1097-0142(20000801)89:3<574::aid-cncr12>3.0.co;2-y

[5] Viale G, Maiorano E, Pruneri G, et al: Predicting the risk for additional axillary metastases in patients with breast carcinoma and positive sentinel lymph node biopsy. Ann Surg 241:319-325, 2005 15650643 10.1097/01.sla.0000150255.30665.52 PMC1356918

[6] Moo TA, Edelweiss M, Hajiyeva S, et al: Is low-volume disease in the sentinel node after neoadjuvant chemotherapy an indication for axillary dissection? Ann Surg Oncol 25:1488-1494, 2018 29572705 10.1245/s10434-018-6429-2 PMC5930130

[7] Barron AU, Hoskin TL, Boughey JC: Predicting non-sentinel lymph node metastases in patients with a positive sentinel lymph node after neoadjuvant chemotherapy. Ann Surg Oncol 25:2867-2874, 2018 29956095 10.1245/s10434-018-6578-3

[8] Weber WP, Matrai Z, Hayoz S, et al: Tailored axillary surgery in patients with clinically node-positive breast cancer: Pre-planned feasibility substudy of TAXIS (OPBC-03, SAKK 23/16, IBCSG 57-18, ABCSG-53, GBG 101). Breast 60:98-110, 2021 34555676 10.1016/j.breast.2021.09.004 PMC8463904

[9] Moo TA, Pawloski KR, Flynn J, et al: Is residual nodal disease at axillary dissection associated with tumor subtype in patients with low volume sentinel node metastasis after neoadjuvant chemotherapy? Ann Surg Oncol 28:6044-6050, 2021 33876362 10.1245/s10434-021-09910-2 PMC10224770

[10] Curigliano G, Burstein HJ, Gnant M, et al: Understanding breast cancer complexity to improve patient outcomes: The St Gallen International Consensus Conference for the primary therapy of individuals with early breast cancer 2023. Ann Oncol 34:970-986, 2023 37683978 10.1016/j.annonc.2023.08.017

[11] Loibl S, Andre F, Bachelot T, et al: Early breast cancer: ESMO clinical practice guideline for diagnosis, treatment and follow-up. Ann Oncol 35:159-182, 2024 38101773 10.1016/j.annonc.2023.11.016

[12] Gradishar WJ, Moran MS, Abraham J, et al: NCCN Guidelines® insights: Breast cancer, version 4.2023. J Natl Compr Canc Netw 21:594-608, 2023 37308117 10.6004/jnccn.2023.0031

[13] Wong SM, Almana N, Choi J, et al: Prognostic significance of residual axillary nodal micrometastases and isolated tumor cells after neoadjuvant chemotherapy for breast cancer. Ann Surg Oncol 26:3502-3509, 2019 31228134 10.1245/s10434-019-07517-2

[14] Boughey JC, Suman VJ, Mittendorf EA, et al: Sentinel lymph node surgery after neoadjuvant chemotherapy in patients with node-positive breast cancer: The ACOSOG Z1071 (alliance) clinical trial. JAMA 310:1455-1461, 2013 24101169 10.1001/jama.2013.278932 PMC4075763

[15] Boileau JF, Poirier B, Basik M, et al: Sentinel node biopsy after neoadjuvant chemotherapy in biopsy-proven node-positive breast cancer: The SN FNAC study. J Clin Oncol 33:258-264, 2015 25452445 10.1200/JCO.2014.55.7827

[16] Weiss A, King C, Vincuilla J, et al: Rates of pathologic nodal disease among cN0 and cN1 patients undergoing routine axillary ultrasound and neoadjuvant chemotherapy. Breast Cancer Res Treat 195:181-189, 2022 35900704 10.1007/s10549-022-06677-2

[17] Burstein HJ, Curigliano G, Thurlimann B, et al: Customizing local and systemic therapies for women with early breast cancer: The St. Gallen International Consensus Guidelines for treatment of early breast cancer 2021. Ann Oncol 32:1216-1235, 2021 34242744 10.1016/j.annonc.2021.06.023 PMC9906308

[18] Srour MK, Tseng J, Luu M, et al: Patterns in the use of axillary operations for patients with node-positive breast cancer after neoadjuvant chemotherapy: A national cancer database (NCDB) analysis. Ann Surg Oncol 26:3305-3311, 2019 31342364 10.1245/s10434-019-07540-3

[19] Boughey JC, Yu H, Dugan CL, et al: Changes in surgical management of the axilla over 11 years—Report on more than 1500 breast cancer patients treated with neoadjuvant chemotherapy on the prospective I-SPY2 trial. Ann Surg Oncol 30:6401-6410, 2023 37380911 10.1245/s10434-023-13759-y PMC11702681

[20] von Elm E, Altman DG, Egger M, et al: The Strengthening the Reporting of Observational Studies in Epidemiology (STROBE) statement: Guidelines for reporting observational studies. Lancet 370:1453-1457, 2007 18064739 10.1016/S0140-6736(07)61602-X

[21] Giuliano AE, Ballman KV, McCall L, et al: Effect of axillary dissection vs no axillary dissection on 10-year overall survival among women with invasive breast cancer and sentinel node metastasis: The ACOSOG Z0011 (alliance) randomized clinical trial. JAMA 318:918-926, 2017 28898379 10.1001/jama.2017.11470 PMC5672806

[22] Galimberti V, Cole BF, Viale G, et al: Axillary dissection versus no axillary dissection in patients with breast cancer and sentinel-node micrometastases (IBCSG 23-01): 10-year follow-up of a randomised, controlled phase 3 trial. Lancet Oncol 19:1385-1393, 2018 30196031 10.1016/S1470-2045(18)30380-2

[23] Savolt A, Peley G, Polgar C, et al: Eight-year follow up result of the OTOASOR trial: The optimal treatment of the axilla—Surgery or radiotherapy after positive sentinel lymph node biopsy in early-stage breast cancer: A randomized, single centre, phase III, non-inferiority trial. Eur J Surg Oncol 43:672-679, 2017 28139362 10.1016/j.ejso.2016.12.011

[24] Wong SM, Weiss A, Mittendorf EA, et al: Surgical management of the axilla in clinically node-positive patients receiving neoadjuvant chemotherapy: A national cancer database analysis. Ann Surg Oncol 26:3517-3525, 2019 31342389 10.1245/s10434-019-07583-6

[25] Kuehn T, Bauerfeind I, Fehm T, et al: Sentinel-lymph-node biopsy in patients with breast cancer before and after neoadjuvant chemotherapy (SENTINA): A prospective, multicentre cohort study. Lancet Oncol 14:609-618, 2013 23683750 10.1016/S1470-2045(13)70166-9

[26] Classe JM, Loaec C, Gimbergues P, et al: Sentinel lymph node biopsy without axillary lymphadenectomy after neoadjuvant chemotherapy is accurate and safe for selected patients: The GANEA 2 study. Breast Cancer Res Treat 173:343-352, 2019 30343457 10.1007/s10549-018-5004-7

[27] Sentinel Lymph Node Biopsy after Neoadjuvant Chemotherapy for Locally Advanced Breast Cancer. https://clinicaltrials.gov/study/NCT03255577 10.1007/BF02984543 15550841

[28] Grabenstetter A, Moo TA, Hajiyeva S, et al: Accuracy of intraoperative frozen section of sentinel lymph nodes after neoadjuvant chemotherapy for breast carcinoma. Am J Surg Pathol 43:1377-1383, 2019 31219817 10.1097/PAS.0000000000001311 PMC6742561

[29] Laws A, Hughes ME, Hu J, et al: Impact of residual nodal disease burden on technical outcomes of sentinel lymph node biopsy for node-positive (cN1) breast cancer patients treated with neoadjuvant chemotherapy. Ann Surg Oncol 26:3846-3855, 2019 31222687 10.1245/s10434-019-07515-4

[30] Weber WP, Matrai Z, Hayoz S, et al: Association of axillary dissection with systemic therapy in patients with clinically node-positive breast cancer. JAMA Surg 158:1013-1021, 2023 37466971 10.1001/jamasurg.2023.2840 PMC10357358

[31] Johnston SRD, Harbeck N, Hegg R, et al: Abemaciclib combined with endocrine therapy for the adjuvant treatment of HR+, HER2-node-positive, high-risk, early breast cancer (monarchE). J Clin Oncol 38:3987-3998, 2020 32954927 10.1200/JCO.20.02514 PMC7768339

[32] Mamounas EF, Bandos, A, White, JR, et al: Loco-regional irradiation in patients with biopsy-proven axillary node involvement at presentation who become pathologically node-negative after neoadjuvant chemotherapy: Primary outcomes of the NRG Oncology/NSABP B-51/RTOG 1304. San Antonio Breast Cancer Symposium, December 5-9, 2023, San Antonio, TX; 2023

[33] Weaver DL, Ashikaga T, Krag DN, et al: Effect of occult metastases on survival in node-negative breast cancer. N Engl J Med 364:412-421, 2011 21247310 10.1056/NEJMoa1008108 PMC3044504

[34] Comparison of Axillary Lymph Node Dissection with Axillary Radiation for Patients with Node-Positive Breast Cancer Treated with Chemotherapy. https://clinicaltrials.gov/ct2/show/record/NCT01901094

[35] Bartels SAL, Donker M, Poncet C, et al: Radiotherapy or surgery of the axilla after a positive sentinel node in breast cancer: 10-year results of the randomized controlled EORTC 10981-22023 AMAROS trial. J Clin Oncol 41:2159-2165, 2023 36383926 10.1200/JCO.22.01565

